# Imaging based artificial intelligence for predicting lymph node metastasis in cervical cancer patients: a systematic review and meta-analysis

**DOI:** 10.3389/fonc.2025.1532698

**Published:** 2025-02-28

**Authors:** Chu-Qian Jiang, Xiu-Juan Li, Zhi-Yi Zhou, Qing Xin, Lin Yu

**Affiliations:** ^1^ Department of Obstetrics, Guangdong Provincial Key Laboratory of Major Obstetric Diseases, Guangdong Provincial Clinical Research Center for Obstetrics and Gynecology, Guangdong-Hong Kong-Macao Greater Bay Area Higher Education Joint Laboratory of Maternal-Fetal Medicine, The Third Affiliated Hospital of Guangzhou Medical University, Guangzhou, China; ^2^ Department of Obstetrics and Gynecology, Guangdong Provincial Key Laboratory of Major Obstetric Diseases, Guangdong Provincial Clinical Research Center for Obstetrics and Gynecology, Guangdong-Hong Kong-Macao Greater Bay Area Higher Education Joint Laboratory of Maternal-Fetal Medicine, The Third Affiliated Hospital of Guangzhou Medical University, Guangzhou, China; ^3^ Department of Clinical Medicine, The Third Clinical School of Guangzhou Medical University, Guangzhou, China

**Keywords:** cervical cancer, lymph node metastasis, artificial intelligence, radiomic, meta-analysis

## Abstract

**Purpose:**

This meta-analysis was conducted to assess the diagnostic performance of artificial intelligence (AI) based on imaging for detecting lymph node metastasis (LNM) among cervical cancer patients and to compare its performance with that of radiologists.

**Methods:**

A comprehensive literature search was conducted across PubMed, Embase, and Web of Science to identify relevant studies published up to October 2024. The search followed the Preferred Reporting Items for Systematic Reviews and Meta-Analyses of Diagnostic Test Accuracy (PRISMA-DTA) guidelines. Studies evaluating the accuracy of AI models in detecting LNM in cervical cancer through computed tomography (CT), magnetic resonance imaging (MRI), and positron emission tomography/computed tomography (PET/CT) were included. Pathology served as the reference standard for validation. A bivariate random-effects model was employed to estimate pooled sensitivity and specificity, both presented alongside 95% confidence intervals (CIs). Bias was assessed with the revised Quality Assessment of Diagnostic Accuracy Studies-2 (QUADAS-2) tool. Study heterogeneity was examined through the I^2^ statistic. Meta-regression was conducted when significant heterogeneity (I^2^ > 50%) was observed.

**Results:**

A total of 23 studies were included in this meta-analysis. The quality and bias of the included studies were acceptable. However, substantial heterogeneity was observed among the included studies. Internal validation sets comprised 23 studies and 1,490 patients. The pooled sensitivity, specificity, and the area under the curve (AUC) for detecting LNM in cervical cancer were 0.83 (95% CI: 0.78-0.87), 0.78 (95% CI: 0.74-0.82) and 0.87 (95% CI: 0.84-0.90), respectively. External validation sets comprised six studies and 298 patients. The pooled sensitivity, specificity, and AUC for detecting LNM were 0.70 (95% CI: 0.56-0.81), 0.85 (95% CI: 0.66-0.95) and 0.76 (95% CI: 0.72-0.79), respectively. For radiologists, eight studies and 644 patients were included; the pooled sensitivity, specificity, and AUC for detecting LNM were 0.54 (95% CI: 0.42-0.66), 0.79 (95% CI: 0.59-0.91) and 0.65 (95% CI: 0.60-0.69), respectively.

**Conclusions:**

Imaging-based AI demonstrates higher diagnostic performance than radiologists. Prospective studies with rigorous standardization as well as further research with external validation datasets, are necessary to confirm the results and assess their practical clinical applicability.

**Systematic Review Registration:**

https://www.crd.york.ac.uk/PROSPERO, identifier CRD42024607074.

## 1 Introduction

Cervical cancer is the fourth most prevalent malignancy among women worldwide, with approximately 604,000 new cases and 342,000 deaths reported each year (1). LNM is a critical prognostic factor that significantly influences survival outcomes. Early-stage patients without LNM have a five-year survival rate ranging from 80% to 100%, while this rate declines markedly to 47% to 78% for those with LNM (2). The International Federation of Gynecology and Obstetrics (FIGO) staging system is the primary framework for guiding treatment and management (3). Patients diagnosed with LNM frequently treated with radiotherapy and chemotherapy as the preferred treatment methods (3). Therefore, early, non-invasive assessment of lymph node status is essential for determining optimal treatment plans and prevent unnecessary surgical interventions, ultimately enhancing patient care and outcomes.

Conventional imaging diagnostic methods, including CT, MRI, and PET/CT, have been commonly used for detecting LNM in cervical cancer. However, these techniques have notable limitations. CT and MRI often exhibit restricted sensitivity and specificity, primarily due to their inability to detect normal-sized LNM smaller than 1 cm, making it difficult to identify micrometastases (4, 5). PET/CT, while offering relatively better performance for LNM detection, faces challenges distinguishing between metastatic and hypermetabolic benign lymph nodes (6, 7). Furthermore, its effectiveness in detecting normal-sized LNM and early-stage LNM is constrained, with sensitivity ranging from only 32% to 58% (8). Although pathological examination is often considered the gold standard for LNM detection due to its accuracy, it is not ideal as an initial diagnostic approach because of its invasive nature, procedural complexity, and associated patient risks.

The emergence of AI has transformed the diagnostic landscape for cervical cancer, particularly in predicting LNM (9). Radiomics, an AI-based technique, extracts numerous quantifiable features from medical imaging data to reveal microstructural characteristics of tumors or other tissues not visible to the naked eye (10). Studies have demonstrated that radiomic features derived from MRI, CT, and PET/CT images are effective in predicting LNM in cervical cancer patients (11–13). However, these studies face contradictions due to small sample sizes, limited cross-comparisons of imaging techniques, and challenges in model reproducibility (14). Furthermore, it remains unclear whether AI-based diagnostics methods can outperform the expertise of experienced radiologists in real-world clinical settings (15).

Thus, we conducted a meta-analysis to evaluate the diagnostic performance of different imaging-based AI methods for LNM in cervical cancer patients, and compared their performance with conventional radiologists.

## 2 Methods

The meta-analysis strictly followed the Preferred Reporting Items for Systematic Reviews and Meta-Analyses of Diagnostic Test Accuracy (PRISMA-DTA) guidelines (16). Additionally, the study protocol is registered with PROSPERO (CRD42024607074).

### 2.1 Search strategy

We performed a comprehensive search throughout PubMed, Embase, and Web of Science databases, completed on October 7, 2024, with an update on November 2, 2024, to ensure the inclusion of recent studies. The search strategy included three primary terms: “artificial intelligence”, “cervical cancer”, and “lymph node metastasis”, applying both keywords and MeSH terms to optimize coverage (**Supplementary Table 1**). Only studies published in English, with accessible full text, were eligible for inclusion. Additionally, reference lists of selected articles were manually reviewed to capture further relevant studies.

### 2.2 Inclusion and exclusion criteria

The inclusion criteria were established based on the PICOS framework. Population (P): Adult cervical cancer patients undergoing LNM evaluation. Intervention (I): Artificial intelligence models utilizing MRI, CT, or PET/CT imaging modalities. Comparison (C): Studies with no comparator or those comparing results with clinicians. Outcome (O): Primary outcomes were sensitivity, specificity, and area under the curve (AUC). Study design (S): Only retrospective and prospective studies were included. Additional criteria required studies to be published in English and to include at least 10 participants.

Exclusion criteria included: (1) irrelevant titles and abstracts; (2) non-eligible publication types, such as reviews, conference abstracts, case reports, and meta-analyses. Studies that did not meet these criteria were excluded to ensure the reliability and quality of data for the meta-analysis.

### 2.3 Quality assessment

Two reviewers conducted independent assessments of bias using a modified quality assessment tool, resolving disagreements through consensus to ensure rigor and objectivity in the evaluation. To enhance the tool’s relevance, we adapted the original QUADAS-2 by incorporating criteria from the PROBAST (Prediction model Risk of Bias Assessment Tool), targeting potential biases unique to AI-based LNM prediction (17, 18). This revised tool focused on four domains: (1) patient selection, (2) index test (AI algorithm), (3) reference standard, and (4) analysis. Applicability concerns were also assessed within the first three domains.

### 2.4 Data extraction

Two reviewers independently assessed study eligibility and conducted data extraction, with any disagreements resolved by consensus involving a third reviewer as an adjudicator for accuracy. Extracted data included (1) study details: first author’s name, publication year, study design, country of origin, and reference standard; (2) patient data: number of patients in training, internal and external validation sets, age distribution, and number of patients with positive LNM; (3) AI algorithm details: imaging modality and algorithm type.

### 2.5 Outcome measures

The primary outcome measures included data from both internal and external validation sets, as well as sensitivity, specificity, and AUC for radiologists. Sensitivity was defined as the ratio of true positives (TP) to the sum of true positives (TP) and false negatives (FN), while specificity was defined as the ratio of true negatives (TN) to the sum of true negatives (TN) and false positives (FP). The AUC, representing the area under the summary receiver operating characteristic (SROC) curve, summarizes the model’s ability to distinguish between positive and negative cases. As a crucial metric for evaluating the accuracy of diagnostic tests, the AUC provides a quantitative measure of performance. Higher values indicate superior diagnostic efficiency and reliability (19). We extracted AI performance data from validation sets, prioritizing the model with the highest AUC. Additionally, radiologists’ diagnostic data were collected for comparative evaluation.

### 2.6 Statistical analysis

We utilized a bivariate random-effects model to estimate pooled sensitivity and specificity for both imaging-based AI and clinician assessments, each reported with 95% CIs. To evaluate diagnostic accuracy, we used SROC model to generate SROC curves and calculate the AUC. The SROC model integrates diagnostic data from multiple studies, illustrating the trade-off between sensitivity and specificity in diagnostic tests. Each point on the curve represents the result of a specific diagnostic test, with its sensitivity and specificity values visually depicted (19). The Fagan plot was used to explain the link between pre-probability, post-probability, and likelihood ratio, which can estimate the application of imaging-based AI in clinical practice (20). Heterogeneity across studies was evaluated using the I^2^ statistic, where values of 0%-25%, 25%-50%, 50%-75%, and >75% signified very low, low, moderate, and high heterogeneity, respectively. For internal validation datasets exceeding 10 studies, meta-regression was conducted when high heterogeneity (I^2^ > 50%) was observed, exploring variables such as imaging type (MRI vs. non-MRI), patient number (>50 vs. ≤50), country (China vs. other countries), and algorithm type (deep learning vs. machine learning). Subgroup analyses were performed for distinct imaging modalities (CT, MRI, and PET/CT).

Publication bias was evaluated using Deeks’ funnel plot asymmetry test, which evaluates bias by examining the symmetry of the funnel plot and performing quantitative analysis (21). Statistical analyses were conducted using Stata 15.1, while study quality was evaluated using RevMan 5.4. Statistical significance was defined as *P* < 0.05.

## 3 Results

### 3.1 Study selection

**Figure 1:**
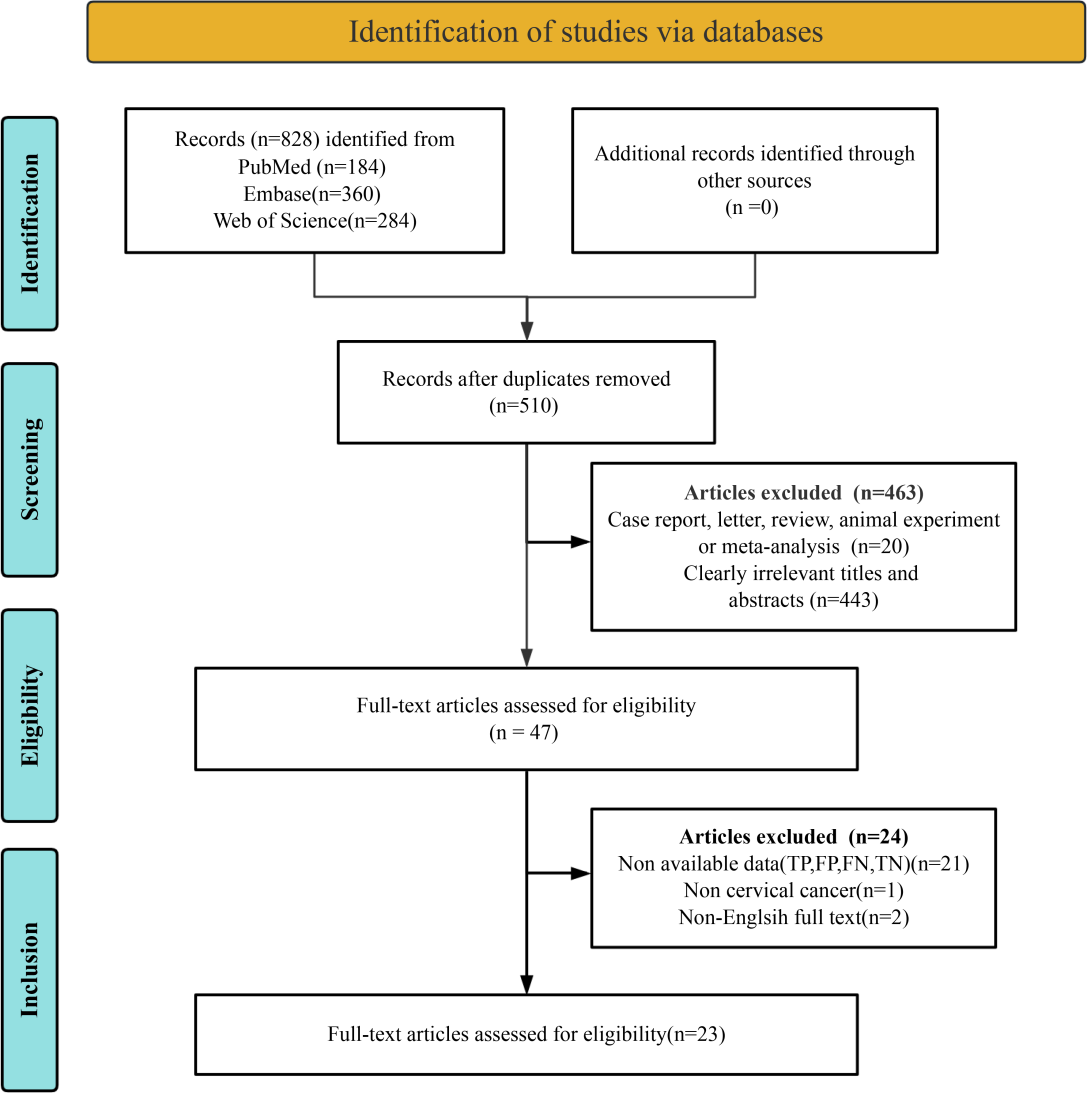
PRISMA flow diagram illustrating the study selection process.

A comprehensive literature search was conducted across three databases. Initially, 828 articles were identified as potentially eligible through the database search. Following the removal of 318 duplicate records, 510 unique articles remained. Of these, 463 studies were excluded due to failure to meet the inclusion criteria. Full-text reviews were conducted on the remaining 47 articles. Subsequently, 24 studies were excluded due to the inability to extract essential data (TP, TN, FP, FN) (n = 21), non-cervical cancer (n = 1), or non-English full text (n = 2). Ultimately, 23 studies were included in the final meta-analysis (9, 11–13, 22–40). The article selection process is illustrated in **Figure 1**, following the PRISMA flow diagram format.

### 3.2 Study description and quality assessment

A total of 23 eligible studies published between 2018 and 2024 were included. The internal validation cohorts across these studies comprised 1,490 patients, with study sample sizes ranging from 28 to 141 participants. Six studies incorporated external validation, involving 298 patients (ranging from 29 to 69) (12, 22, 26, 29, 31, 32); however, one study lacked sufficient data for extraction (22). Eight studies provided comparisons with radiologists, including 644 patients (ranging from 29 to 141) (9, 13, 24–26, 32, 34, 37). All studies were retrospective. The imaging modalities used were predominantly MRI (17 studies) (9, 11, 22–25, 28, 30–37, 39, 40), followed by PET/CT (4 studies) (13, 27, 29, 38), and contrast-enhanced CT (2 studies) (12, 26). Pathology was employed as the reference standard. A summary of patient characteristics is presented in **Table 1**.

**Table 1:** Study and patient characteristics of the included studies.

Author	Year	Country	Study design	Imaging modality	Reference standard	patients per set			Age (Mean ± SD)	No. of LNM+ patients
						Training	Internal validation	External validation		
Wang et al. (11)	2024	China	Retro	MRI	Pathology	86	38	NA	Training: Mean (Quartile): LNM: 56 (Q1:50; Q3:60); non-LNM: 56 (Q1:48; Q3: 60) Internal validation: Mean ± SD: 52.7 ± 8.6; non LNM: 58.6 ± 11.3	Training: 22 Internal validation: 15
Ai et al. (22)	2023	China	Retro	MRI	Pathology	162	68	56	Training: Mean ± SD: LNM: 45.8 ± 10.3; non-LNM: 47.1 ± 8.6 Internal validation: Mean ± SD: LNM: 45.9 ± 8.5; non LNM: 47.5 ± 8.9 External validation: Mean ± SD: LNM: 47.8 ± 3; non LNM: 47.3 ± 9	Training: 38 Internal validation: 16 External validation: 14
Liu et al. (12)	2021	China	Retro	Contrast-enhance CT	Pathology	148	74	51	NA	Training: 48 Internal validation: 23 External validation: 5
Li et al. (26)	2023	China	Retro	Contrast-enhance CT	Pathology	296	122	62	Training: Mean ± SD: LNM: 47.58 ± 7.93; non-LNM: 48.96 ± 9.33 Internal validation: Mean ± SD: LNM: 47.0 ± 7.65; non LNM: 48.97 ± 8.81 External validation: Mean ± SD: LNM: 47.20 ± 8.687; non LNM: 48.04 ± 7.348	Training: 87 Internal validation: 32 External validation: 15
Wu et al. (9)	2020	China	Retro	MRI	Pathology	338	141	NA	Training: Mean ± SD: LNM: 48.8 ± 10.0; non-LNM: 49.9 ± 9.5 Internal validation: Mean ± SD: LNM: 47.6 ± 9.1; non LNM: 48.0 ± 10.2	Training: 71 Internal validation: 32
Deng et al. (23)	2020	China	Retro	MRI	Pathology	89	45	NA	Training: Mean ± SD: LNM: 48.4 ± 7.9; non-LNM: 49.9 ± 8.1 Internal validation: Mean ± SD: LNM: 49.1 ± 8.6; non LNM: 50.2 ± 7.7	Training: 33 Internal validation: 17
Zhang et al. (39)	2022	China	Retro	MRI	Pathology	89	45	NA	Training: Mean ± SD: LNM: 52.39 ± 8.47; non-LNM: 50.63 ± 8.50 Internal validation: Mean ± SD: LNM: 52.44 ± 10.60; non LNM: 51.85 ± 8.06	Training: 69 Internal validation: 35
Song et al. (33)	2021	China	Retro	MRI	Pathology	90	42	NA	Training: Mean ± SD: LNM: 46.83 ± 8.22; non-LNM: 45.10 ± 9.15 Internal validation: Mean ± SD: LNM: 48.95 ± 7.25; non LNM: 47.13 ± 6.83	Training: 65 Internal validation: 29
Yu et al. (38)	2024	China	Retro	PET/CT	Pathology	122	65	NA	Training (95% CI): 56 (23–77) Internal validation (95% CI): 50 (29–68)	Training: 42 Internal validation: 11
Zhang et al. (40)	2023	China	Retro	MRI	Pathology	172	75	NA	Training: Mean ± SD: LNM: 51.80 ± 11.68; non-LNM: 54.91 ± 8.79 Internal validation: Mean ± SD: LNM: 55.54 ± 10.77; non LNM: 54.04 ± 10.23	Training: 55 Internal validation: 24
Xiao et al. (37)	2022	China	Retro	MRI	Pathology	72	32	NA	Training: Mean ± SD: 47.5 ± 12.0 Internal validation: Mean ± SD: 46.6 ± 9.8	Training: 22 Internal validation: 10
Shi et al. (32)	2021	China	Retro	MRI	Pathology	93	47	29	Training: Mean ± SD: LNM: 49.16 ± 9.37; non-LNM: 50.50 ± 9.11 Internal validation: Mean ± SD: LNM: 49.53 ± 9.97; non LNM: 50.43 ± 8.94 External validation: Mean ± SD: LNM: 53.67 ± 7.39; non LNM: 55.00 ± 6.68	Training: 37 Internal validation: 19 External validation: 12
Lucia et al. (29)	2023	France	Retro	PET/CT	Pathology	102	76	31	Training: Median (range): 51 (29–79) Internal validation: Median (range): 52 (26–77) External validation: Median (range): 51 (29–70)	Training: 18 Internal validation: 16 External validation: 5
Xiao et al. (36)	2020	China	Retro	MRI	Pathology	155	78	NA	Training: Mean ± SD: 49.29 ± 9.83 Internal validation: Mean ± SD: 51.30 ± 9.60	Training: 50 Internal validation: 32
Li et al. (27)	2021	China	Retro	PET/CT	Pathology	69	28	NA	Training: Median (range): 52 (33–74) Internal validation: Median (range): 48 (38–65)	Training: 25 Internal validation: 14
Qin et al. (31)	2024	China	Retro	MRI	Pathology	225	98	69	Training: LNM: ≥50: 48; <50: 27; non-LNM: ≥50: 94; <50: 56 Internal validation: LNM: ≥50: 17; <50: 16; non-LNM: ≥50: 36; <50: 29 External validation: LNM: ≥50: 12; <50: 8; non-LNM: ≥50: 38; <50: 11	Training: 75 Internal validation: 33 External validation: 20
Yang et al. (13)	2023	China	Retro	PET/CT	Pathology	135	58	NA	LNM: ≥50: 27; <50: 27; non-LNM: ≥50: 31; <50: 21	Training: 58 Internal validation: 42
Kan et al. (25)	2018	China	Retro	MRI	Pathology	100	43	NA	Training: Mean ± SD: LNM: 49.11 ± 10.09; non-LNM: 51.50 ± 8.89 Internal validation: Mean ± SD: LNM: 51.57 ± 9.92; non LNM: 49.10 ± 8.35	Training: 44 Internal validation: 14
Liu et al. (28)	2024	China	Retro	MRI	Pathology	171	111	NA	Training: LNM: >50: 18; ≤50: 25; non-LNM: >80: 94; ≤50: 48 Internal validation: LNM: >50: 21; ≤50: 7; non-LNM: >50: 62; ≤50: 21	Training: 43 Internal validation: 28
Wu et al. (34)	2019	China	Retro	MRI	Pathology	126	63	NA	Training: Mean (range): LNM: 49 (33–67); non-LNM: 50 (27–71) Internal validation: Median (range): LNM: 48 (29–67); non-LNM: 50 (32–75)	Training: 35 Internal validation: 14
Hou et al. (24)	2020	China	Retro	MRI	Pathology	115	53	NA	Training: Mean ± SD: LNM: 49.86 ± 7.68; non-LNM: 52.10 ± 9.87 Internal validation: Mean ± SD: LNM: 52.36 ± 7.89; non LNM: 53.14 ± 12.44	Training: 28 Internal validation: 11
Xia et al. (35)	2022	China	Retro	MRI	Pathology	104	45	NA	Training: Mean: LNM: 47.12; non-LNM: 46.66 Internal validation: Mean: LNM: 43.30; non-LNM: 46.60	Training: 25 Internal validation: 10
Qian et al. (30)	2022	China	Retro	MRI	Pathology	126	43	NA	Training: Mean ± SD: LNM: 52.57 ± 9.54; non-LNM: 50.45 ± 10.59 Internal validation: Mean ± SD: LNM: 50.64 ± 7.19; non LNM: 53.16 ± 9.73	Training: 25 Internal validation: 10

Retro retrospective; MRI, magnetic resonance imaging; CT, computed tomography; PET, positron emission tomography; LNM, lymph node metastasis; NA, not available.

**Figure 2:**
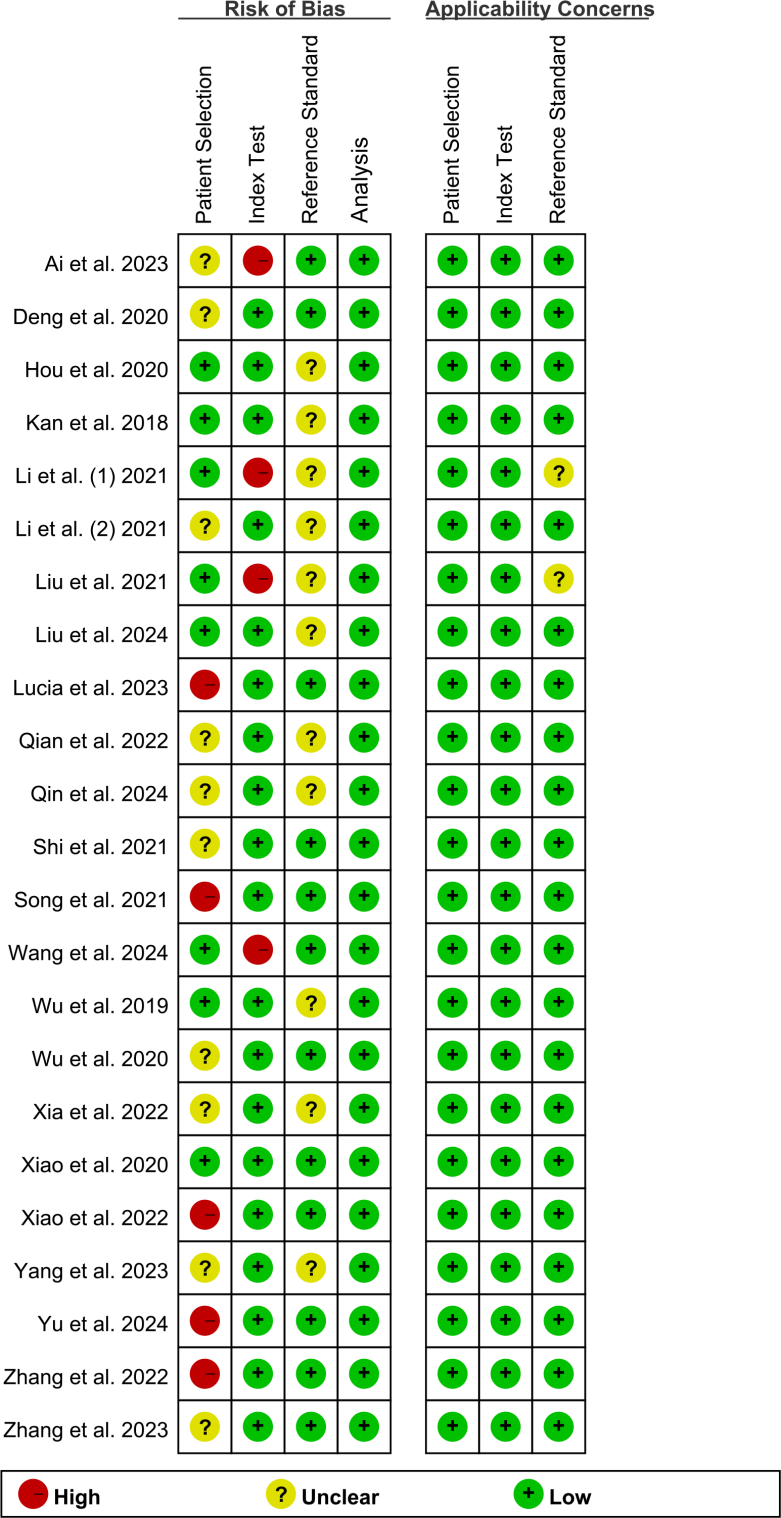
Risk of bias and applicability concerns of the included studies using the Quality Assessment of Diagnostic Performance Studies QUADAS-2 revised tool.

Bias was evaluated using the QUADAS-2-Revised tool, with individual risk assessments illustrated in **Figure 2**. Five studies were rated as having a “high risk” for patient selection due to inappropriate exclusions (29, 33, 36, 38, 39). Four studies were identified as “high risk” for the index test due to inadequate details regarding the artificial intelligence model (11, 12, 22, 26). Overall, despite some areas of concern, the quality of the included studies was deemed acceptable.

### 3.3 Diagnostic performance of internal validation set for AI and radiologists in predicting lymph node metastasis of cervical cancer

**Figure 3:**
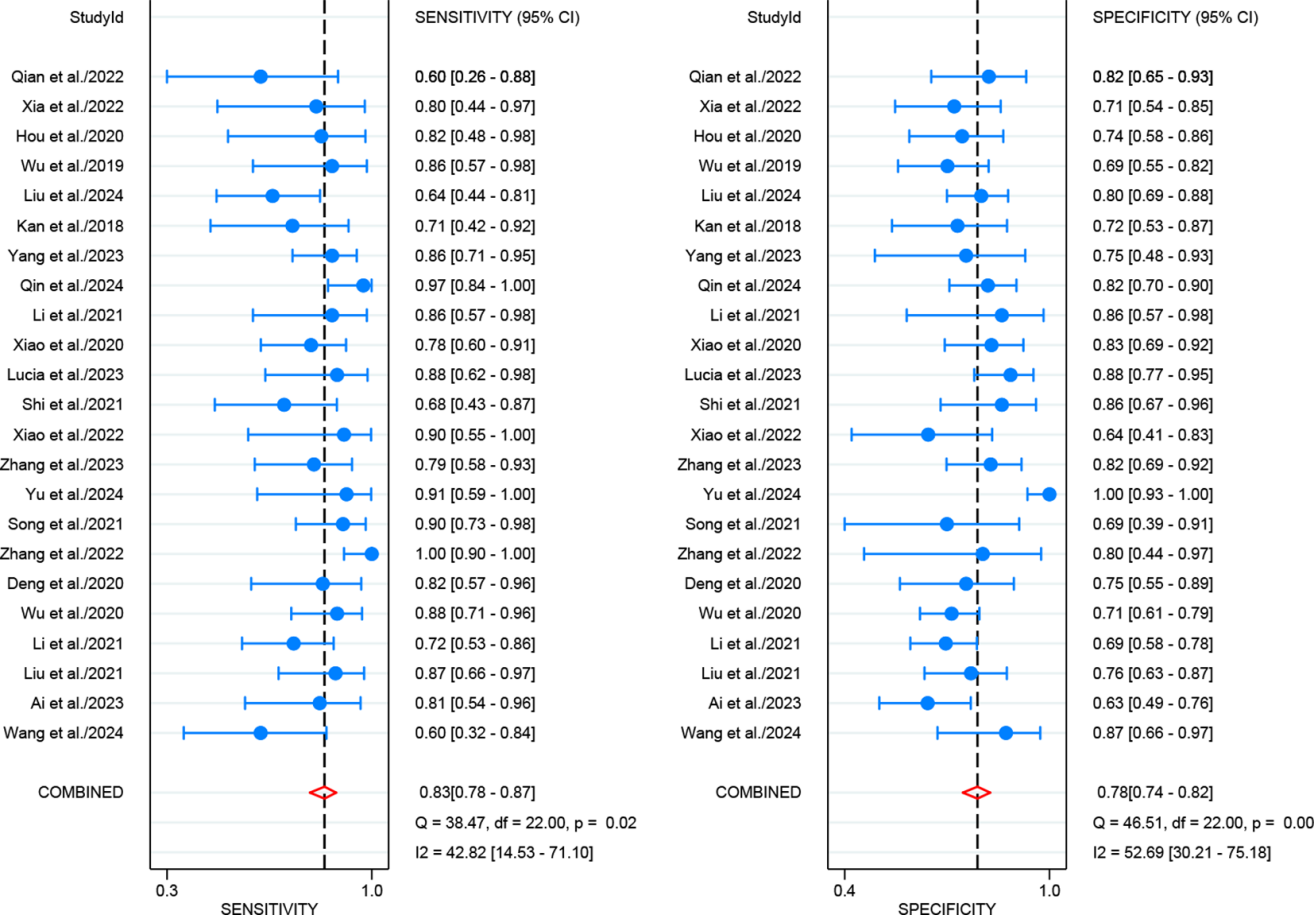
Forest plot of imaging-based artificial intelligence on the internal validation set for diagnosing lymph node metastasis in cervical cancer.

**Figure 4:**
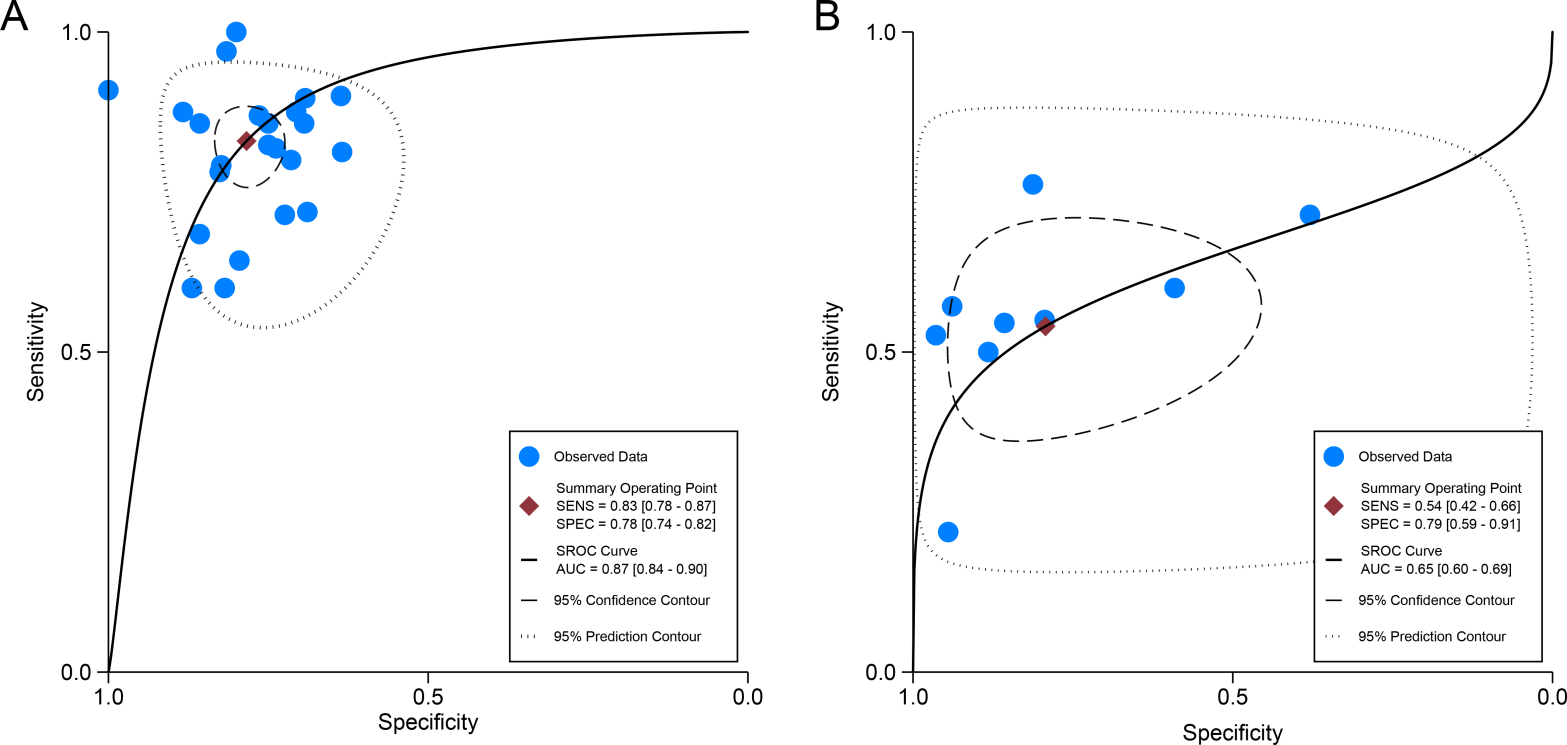
Summary receiver operating characteristic (SROC) curves of imaging-based artificial intelligence on the internal validation set **(A)** and radiologists **(B)** for diagnosing lymph node metastasis in cervical cancer.

**Figure 5:**
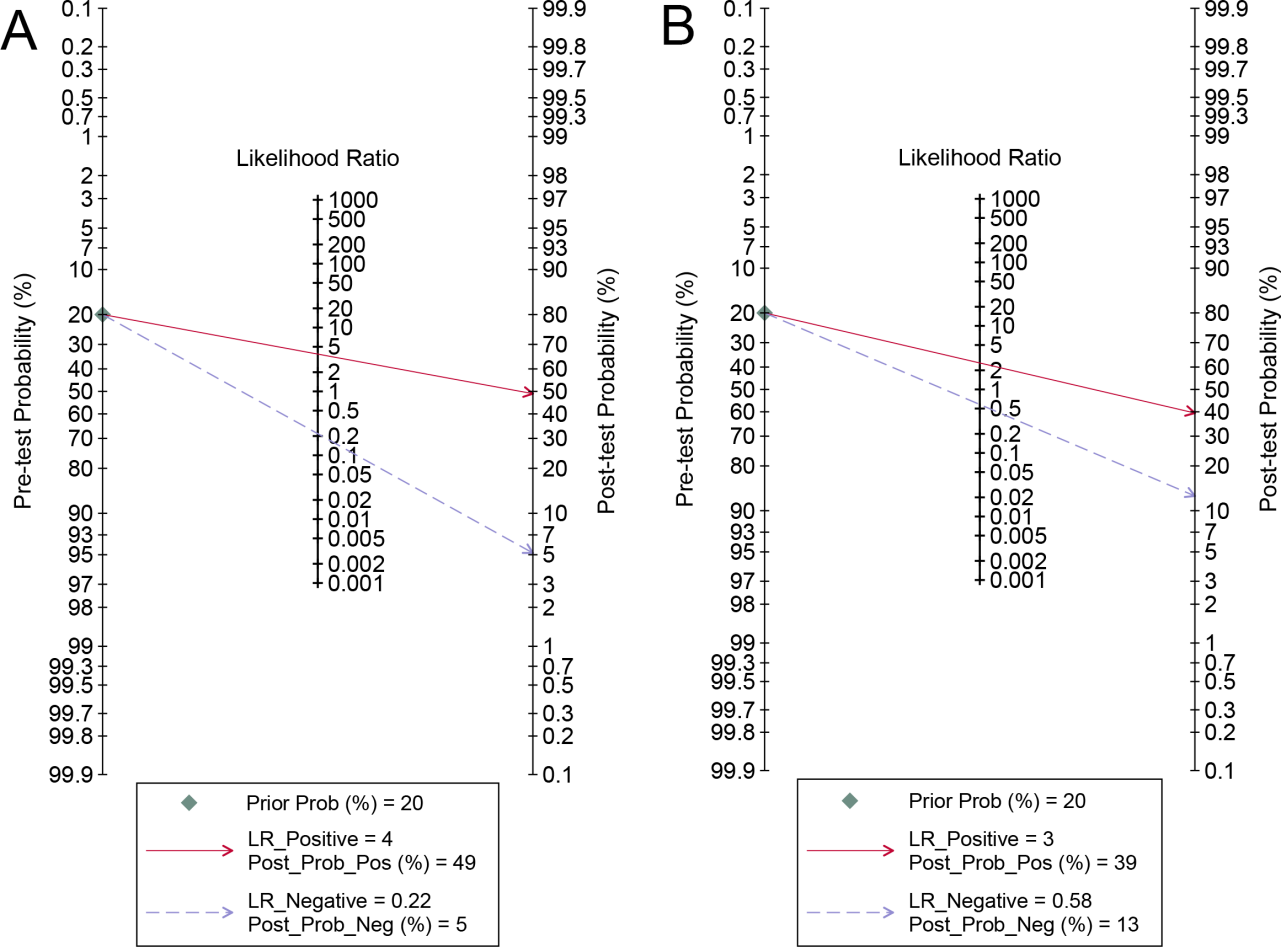
Fagan’s nomogram of imaging-based artificial intelligence on the internal validation set **(A)** and radiologists **(B)** for diagnosing lymph node metastasis in cervical cancer.

**Figure 6:**
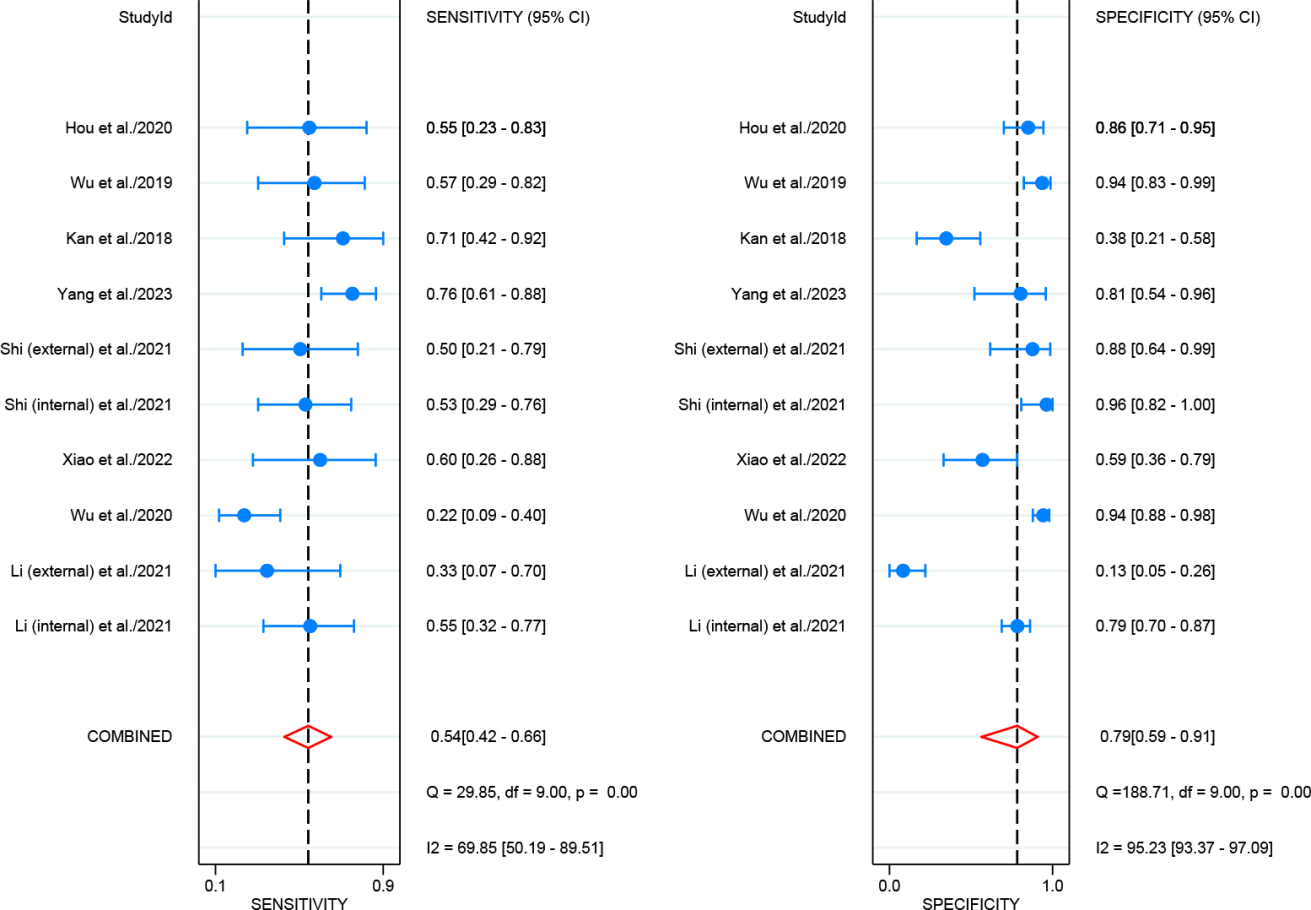
Forest plot of radiologists for diagnosing lymph node metastasis in cervical cancer.

For internal validation sets, the pooled sensitivity and specificity for detecting LNM in cervical cancer were 0.83 (95% CI: 0.78-0.87) and 0.78 (95% CI: 0.74-0.82), respectively (**Figure 3**), with an AUC of 0.87 (95% CI: 0.84-0.90) (**Figure 4A**). With a pre-test probability of 20%, the Fagan nomogram indicates a positive likelihood ratio of 49% and a negative likelihood ratio of 5% (**Figure 5A**). For radiologists, the sensitivity and specificity for detecting LNM in cervical cancer were 0.54 (95% CI: 0.42-0.66) and 0.79 (95% CI: 0.59-0.91), respectively (**Figure 6**), with an AUC of 0.65 (95% CI: 0.60-0.69) (**Figure 4B**). Using the same pre-test probability, the Fagan nomogram indicates a positive likelihood ratio of 39% and a negative likelihood ratio of 13% (**Figure 5B**). The overall diagnostic performance of internal validation, external validation, and radiologists is summarized in **Table 2**.

**Table 2:** Diagnostic performance of internal validation set, external validation set, and radiologists.

Cohort	Studies, n	Sensitivity(95%CI)	I^2^(%)	Specificity(95%CI)	I^2^(%)	AUC(95%CI)
Internal validation set	23	0.83 (0.78; 0.87)	42.82	0.78 (0.74; 0.82)	52.69	0.87 (0.84; 0.90)
External validation set	6	0.70 (0.56; 0.81)	26.22	0.85 (0.66; 0.95)	80.97	0.76 (0.72; 0.79)
Radiologists	8	0.54 (0.42; 0.66)	69.85	0.79 (0.59; 0.91)	95.23	0.65 (0.60; 0.69)

AUC, area under curve.

For internal validation sets, moderate heterogeneity was observed for both sensitivity (I^2^ = 43%) and specificity (I^2^ = 53%). Meta-regression analysis indicated that heterogeneity was primarily due to differences in the number of patients (>50 vs. ≤50, *P* = 0.01 for sensitivity, *P* < 0.001 for specificity). Additionally, algorithm type (deep learning vs. machine learning, *P* = 0.03 for sensitivity, *P* < 0.001 for specificity) and imaging modality (MRI vs. non-MRI, *P* = 0.03 for sensitivity, *P* = 0.01 for specificity) were also identified as potential sources of heterogeneity for both sensitivity and specificity. These findings are further detailed in **Table 3**.

**Table 3:** Subgroup analysis and meta-regression analysis.

Covariate	Studies, n	Sensitivity (95%CI)	*P*-value	Specificity (95%CI)	*P*-value
Number of patients included			0.01		0.00
>50	13	0.84 (0.78; 0.89)		0.79 (0.74; 0.84)	
≤50	10	0.82 (0.74; 0.90)		0.78 (0.71; 0.85)	
Country			0.74		0.86
China	22	0.83 (0.78; 0.87)		0.78 (0.74; 0.82)	
France	1	0.88 (0.69; 1.00)		0.89 (0.78; 0.99)	
Algorithm type			0.03		0.00
Deep learning	4	0.83 (0.73; 0.94)		0.75 (0.66; 0.84)	
Machine learning	19	0.83 (0.78; 0.88)		0.79 (0.75; 0.84)	
Imaging			0.03		0.01
MRI	17	0.82 (0.77; 0.88)		0.76 (0.72; 0.81)	
Non-MRI	6	0.85 (0.76; 0.93)		0.84 (0.77; 0.90)	

MRI, magnetic resonance imaging.

### 3.4 Diagnostic performance of external validation sets for AI in predicting lymph node metastasis in cervical cancer

For external validation sets, the pooled sensitivity and specificity for detecting LNM in cervical cancer were 0.70 (95% CI: 0.56-0.81) and 0.85 (95% CI: 0.66-0.95) (**Supplementary Figure 1**), with an AUC of 0.76 (95% CI: 0.72-0.79) (**Supplementary Figure 2**). With a pre-test probability of 20%, the Fagan nomogram demonstrates a positive likelihood ratio of 55% and a negative likelihood ratio of 8% (**Supplementary Figure 3**).

### 3.5 Diagnostic performance of different imaging techniques for AI in predicting lymph node metastasis in cervical cancer

For MRI-based AI, 17 studies in internal validation were pooled, the sensitivity in detecting LNM of cervical cancer was 0.82 (95% CI: 0.78-0.86), and the specificity was 0.76 (95% CI: 0.72-0.79), with an AUC of 0.85 (95% CI: 0.81-0.88) (**Table 4**). Two studies in external validation were pooled; the sensitivity in detecting LNM of cervical cancer was 0.59 (95% CI: 0.41-0.76), and the specificity was 0.86 (95% CI: 0.76-0.94) (**Table 4**).

**Table 4:** Subgroup analysis based on different AI imaging techniques.

	Interval validation				External validation			
Imaging	Studies, n	Sensitivity (95%CI)	Specificity (95%CI)	AUC (95%CI)	Studies, n	Sensitivity (95%CI)	Specificity (95%CI)	AUC (95%CI)
MRI	17	0.82 (0.78;0.86)	0.76 (0.72;0.79)	0.85 (0.81;0.88)	2	0.59 (0.41;0.76)	0.86 (0.76;0.94)	NA
PET/CT	4	0.87 (0.78;0.93)	0.91 (0.85;0.95)	0.93 (0.88;0.97)	1	NA	NA	NA
CT	2	0.78 (0.65;0.88)	0.72 (0.63;0.79)	NA	2	0.80 (0.56; 0.94)	0.68 (0.57; 0.77)	NA

MRI, magnetic resonance imaging; CT, computed tomography; PET, positron emission tomography; AUC, area under curve; NA, not available.

For PET/CT-based AI, four studies in internal validation were pooled, the sensitivity in detecting LNM of cervical cancer was 0.87 (95% CI: 0.78-0.93), and the specificity was 0.91 (95% CI: 0.85-0.95), with an AUC of 0.93 (95% CI: 0.88-0.97) (**Table 4**). However, an analysis of the external validation set could not be performed owing to the unavailability of sufficient data.

For CT-based AI, two studies in internal validation were pooled; the sensitivity in detecting LNM of cervical cancer was 0.78 (95% CI: 0.65-0.87), and the specificity was 0.72 (95% CI: 0.63-0.79) (**Table 4**). Two studies in external validation were pooled, the sensitivity in detecting LNM of cervical cancer was 0.80 (95% CI: 0.56-0.94), and the specificity was 0.68 (95% CI: 0.57-0.77) (**Table 4**).

### 3.6 Publication bias

**Figure 7:**
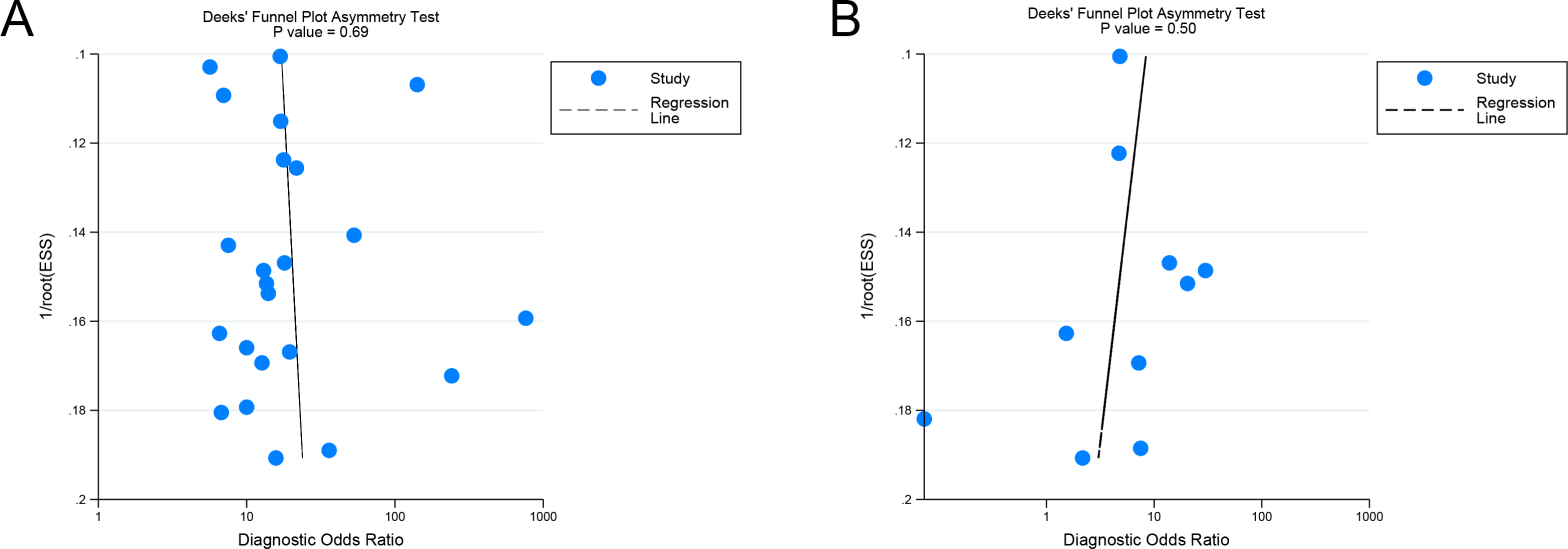
Deek’s funnel plot of imaging-based artificial intelligence on the internal validation set **(A)** and radiologists **(B)** for diagnosing lymph node metastasis in cervical cancer.

Deeks’ funnel plot asymmetry test indicated no significant publication bias for the internal validation sets for AI and radiologists (*P* = 0.69, 0.50) (**Figures 7A, B**). Similarly, no significant publication bias was identified for the external validation sets (P = 0.18) (**Supplementary Figure 4**).

## 4 Discussion

In recent years, advancements in AI have led to the development of models aimed at assessing LNM in cervical cancer using imaging techniques, incorporating technologies such as MRI, CT, and PET/CT (12, 25, 29). However, despite this promising integration, the diagnostic performance of imaging-based AI compared with radiologists, has demonstrated variability across studies. For instance, research by Kan et al. highlighted that MRI-based AI models exhibited superior diagnostic accuracy for detecting LNM in cervical cancer compared to traditional radiologist assessments, suggesting the potential for enhanced precision through AI implementation (25). Conversely, findings from other studies, such as those by Shi et al., indicated that MRI-based AI models did not surpass the specificity achieved by experienced clinical radiologists, reflecting inconsistencies in diagnostic outcomes (32). These discrepancies underscore the need for further investigation into the comparative diagnostic efficacy of AI and human radiologists in this domain.

This study presents a meta-analysis of the diagnostic performance of imaging-based AI in detecting LNM in cervical cancer. Our findings demonstrate that AI models in internal validation cohorts achieved higher sensitivity (0.83 versus 0.54) and AUC (0.87 versus 0.65) compared to radiologists while maintaining comparable specificity (0.79 versus 0.78). The higher sensitivity and AUC of AI models may be attributed to their ability to detect subtle imaging features that may be overlooked by human observers, enhancing diagnostic accuracy (14). Radiologists demonstrate specificity comparable to that of AI models, possibly due to their ability to utilize clinical context and experiential judgment, which helps to reduce false-positive results (41). Overall, imaging-based AI exhibits enhanced diagnostic performance, particularly in accurately identifying patients with LNM.

Subgroup analysis of different imaging modalities in internal validation revealed that MRI, PET/CT, and CT demonstrated sensitivities of 0.82, 0.87, and 0.78, respectively, with specificities of 0.76, 0.91, and 0.72. The AUC values were 0.85 for MRI and 0.93 for PET/CT, while the AUC for CT could not be evaluated due to insufficient data. Our findings indicate that PET/CT-based AI showed superior diagnostic performance compared to MRI and CT. This enhanced performance is likely due to the integration of metabolic information with anatomical imaging in PET/CT and its ability to extract high-throughput imaging features reflecting metabolic characteristics (27, 42). In contrast, MRI-based and CT-based AI primarily rely on anatomical features alone.

This meta-analysis represents the first effort to evaluate the diagnostic performance of imaging-based AI models and directly compare their performance with that of radiologists in predicting LNM in cervical cancer patients. A meta-analysis by He et al. on traditional imaging techniques (MRI vs. PET/CT) reported a sensitivity of 0.65 (0.60–0.69) and specificity of 0.93 (0.91–0.94) for PET/CT, along with a sensitivity of 0.58 (0.54–0.63) and specificity of 0.91 (0.90–0.92) for MRI (43). Compared with the internal validation results of our study, their findings show lower sensitivity. These results are consistent with our comparison of imaging-based AI models and radiologists, further demonstrating the robustness of our findings.

Our study introduces an innovative approach by incorporating both internal and external validation datasets to evaluate the generalizability and reliability of AI models. A previous meta-analysis by Li et al., which evaluated the diagnostic performance of MRI-based AI in detecting lymph node metastasis in cervical cancer, reported a sensitivity of 0.80, specificity of 0.76, and AUC of 0.83 (44), results that are closely aligned with our findings for MRI-based AI models. However, we extended the scope beyond MRI to include multiple imaging modalities (CT and PET/CT). This broader approach offers novel and clinically relevant insights into AI applications in diverse imaging methods, providing actionable strategies for optimizing diagnostic workflows.

Imaging-based AI models present significant advantages in predicting LNM in cervical cancer patients, particularly due to their higher sensitivity compared to traditional methods, which can enhance detection performance. Our results demonstrates that AI achieves superior diagnostic performance (AUC: 0.87 versus 0.65 for radiologists), suggesting its potential to reduce healthcare providers’ workload and enhance patient outcomes through early detection and timely intervention. Notably, PET/CT-based AI showed superior diagnostic performance, warranting future studies to compare AI models across different imaging modalities.

The clinical significance of imaging-based AI lies in its ability to enable rapid detection and its high acceptance among patients. Although previous studies have explored other invasive methods for diagnosing and treating early cervical cancer patients, concerns regarding surgical trauma and complications remain substantial (45). Integrating imaging-based AI with these methods represents a potential direction for future clinical practice. Additionally, the study by Mereu et al. on locally advanced cervical cancer demonstrates that the treatment approach combining neoadjuvant chemotherapy with radical surgery shows limited effectiveness for patients with LNM, as it does not result in significant improvements in disease-free survival or overall survival (46). Consequently, the early detection of LNM in cervical cancer is crucial in clinical practice to prevent unnecessary surgery and chemotherapy and to develop appropriate strategies, such as precise resection or radiotherapy (12). However, the high heterogeneity of our results highlights the need for further research and external validation to confirm their robustness.

Several limitations of this meta-analysis must be considered when interpreting the results. First, the high heterogeneity among the included studies may have affected the overall sensitivity and specificity of AI models in both internal and external datasets. Meta-regression identified patient numbers, algorithm types, and imaging modalities as potential sources of heterogeneity. Notably, heterogeneity may also be attributed to variations in study design methodologies, patient demographic characteristics, tumor staging criteria, institutional imaging protocols, image acquisition parameters, and differences in radiologist experience levels and training backgrounds. Additionally, to reduce variability in the research, our study focused exclusively on imaging-based AI models and did not evaluate AI models incorporating other factors, such as clinical variables. The primary objective was to assess LNM detection in cervical cancer; other pathological factors, such as lymphovascular space invasion (LVSI), were not included in this analysis. Although some studies have explored the diagnostic performance of these pathological factors, integrating them into a comprehensive analysis remains a critical avenue for future research (47). Second, all included studies were retrospective, which introduces potential biases. Well-designed prospective studies with external datasets are necessary to validate our findings. Additionally, the majority of the studies were from China, which may also contribute to potential bias. Third, external validation was insufficient. Only six of the 23 studies included external testing. External validation is critical to address overfitting, a common issue in AI development, where models perform well on internal data but may underperform on external datasets. This discrepancy underscores the importance of following AI development guidelines that emphasize external validation before clinical application (48). Future research should prioritize rigorous external validation to ensure the durability and practical utility of AI algorithms in real-world clinical applications.

## 5 Conclusion

Imaging-based AI demonstrates higher diagnostic performance than radiologists. Prospective studies with rigorous standardization as well as further research with external validation datasets, are necessary to confirm the results and assess their practical clinical applicability.

## Data Availability

The original contributions presented in the study are included in the article/**Supplementary Material**. Further inquiries can be directed to the corresponding author.
