# Supplementary material for: Imaging based artificial intelligence for predicting lymph node metastasis in cervical cancer patients: a systematic review and meta-analysis
Source: Front Oncol. 2025 Feb 28;15:1532698. doi: 10.3389/fonc.2025.1532698 (PMC11906327; doi:10.3389/fonc.2025.1532698)

Supplementary materials

Supplementary Table 1 Search strategy in PubMed, Embase and Web of Science.

| Database | Search strategy |
| --- | --- |
| PubMed | ("Radiomics"[Mesh] OR "Artificial Intelligence"[Mesh] OR "Machine Learning"[Mesh] OR "Deep Learning"[Mesh] OR "Artificial Intelligence"[Title/Abstract] OR "AI"[Title/Abstract] OR "Machine Learning"[Title/Abstract] OR "Deep Learning"[Title/Abstract] OR “Machine Intelligence”[Title/Abstract] OR “Radiomic”[Title/Abstract]) AND ("Uterine Cervical Neoplasms"[Mesh] OR “cervical”[Title/Abstract] OR “cervix”[Title/Abstract] OR “Cervix Cancer”[Title/Abstract] OR “Cervical Cancer”[Title/Abstract] OR “Cervix Neoplasms” [Title/Abstract]) AND ("Lymphatic Metastasis"[Mesh] OR “Lymphatic Metastases”[Title/Abstract] OR “Lymph Node Metastasis”[Title/Abstract] OR “LNM”[Title/Abstract] OR “node Metastasis”[Title/Abstract] OR “Lymph Node Metastases”[Title/Abstract]) |
| Embase | ('radiomics'/exp OR 'artificial intelligence'/exp OR 'machine learning'/exp OR 'deep learning'/exp OR ‘Artificial Intelligence’:ab,ti OR ‘AI’:ab,ti OR ‘Machine Learning’:ab,ti OR ‘Deep Learning’:ab,ti OR ‘Machine Intelligence’:ab,ti OR ‘Radiomic’:ab,ti) AND ('uterine cervix tumor'/exp OR ‘cervical’:ab,ti OR ‘cervix cancer’:ab,ti OR ‘cervical cancer’:ab,ti OR ‘cervical neoplasia’:ab,ti OR ‘cervix neoplasia’:ab,ti) AND ('lymph node metastasis'/exp OR 'Lymph Node Metastasis':ab,ti OR 'LNM':ab,ti OR 'node Metastasis':ab,ti) |
| Web of Science | ((TS=("Radiomics" OR "Artificial Intelligence" OR "Machine Learning" OR "Deep Learning" OR "AI" OR "Machine Learning" OR "Deep Learning" OR “Machine Intelligence”)) AND TS=("Lymphatic Metastasis" OR "Lymph Node Metastasis" OR " LNM" OR “node Metastasis”)) AND TS=("Uterine Cervical Neoplasms" OR “cervical” OR “cervix” OR “Cervix Cancer” OR “Cervical Cancer”) |

Supplementary Figure 1 Forest plot of imaging-based artificial intelligence on the external validation set for diagnosing lymph node metastasis in cervical cancer.


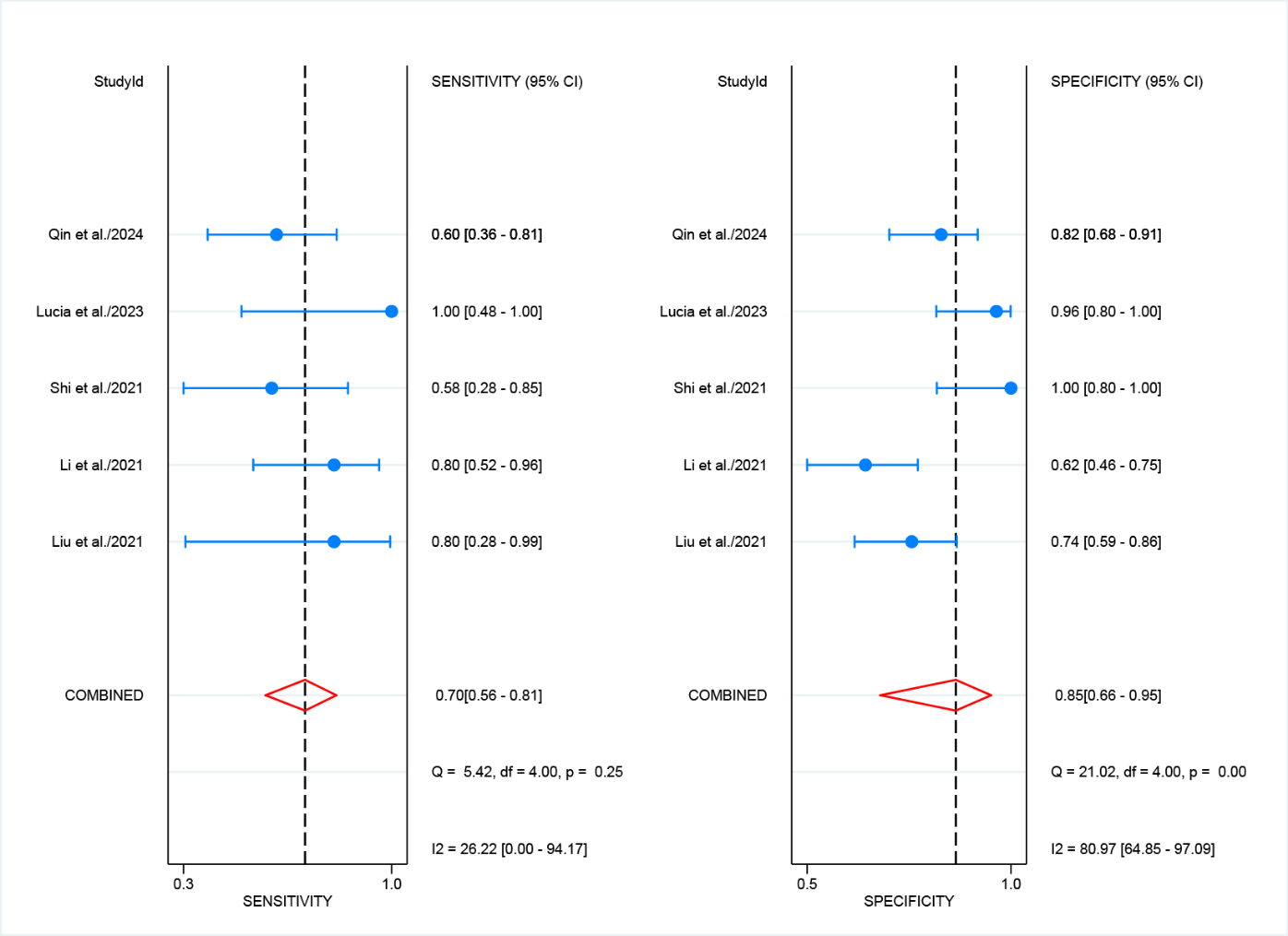


Supplementary Figure 2 Summary receiver operating characteristic (SROC) curves of imaging-based artificial intelligence on the external validation set for diagnosing lymph node metastasis in cervical cancer.


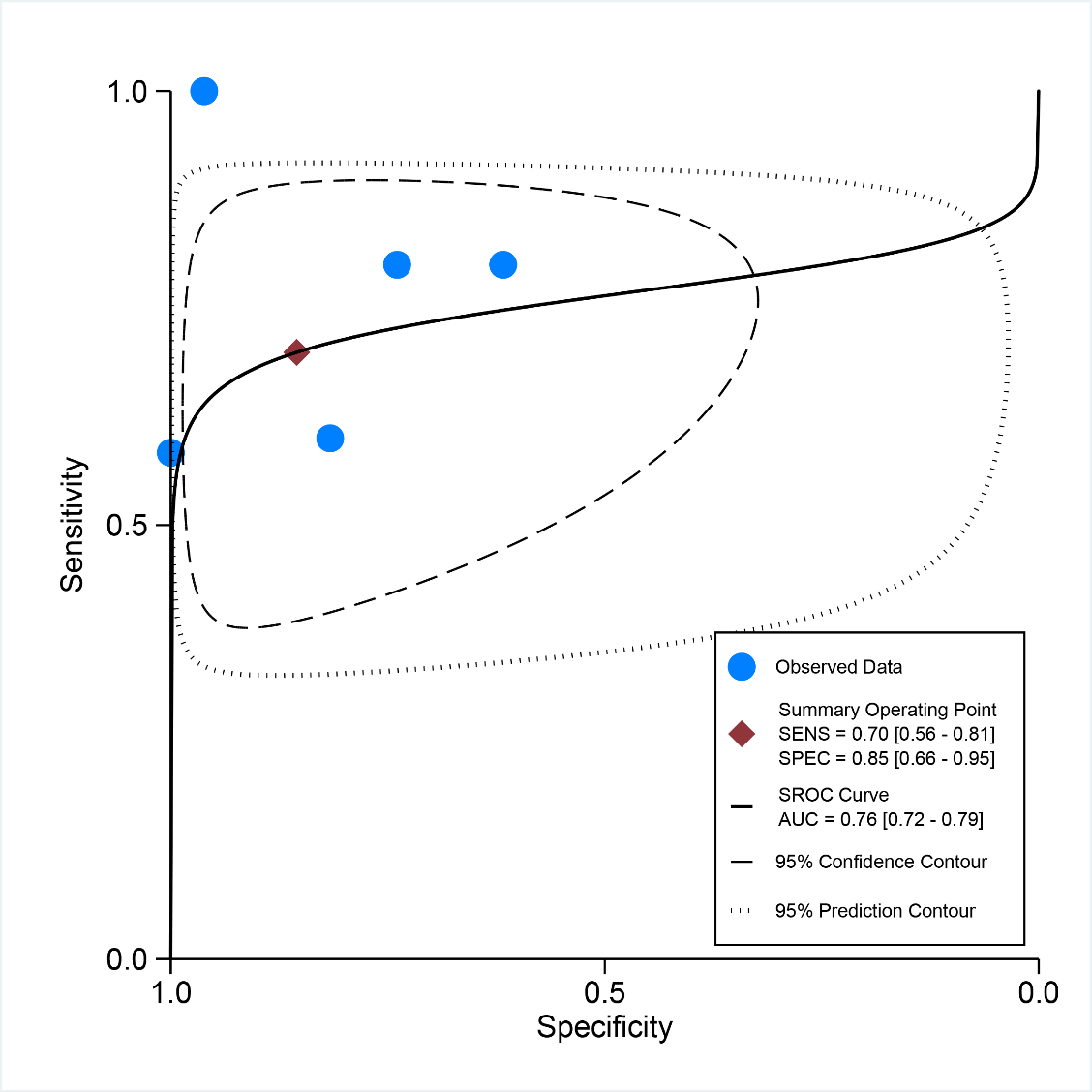


Supplementary Figure 3 Fagan's nomogram of imaging-based artificial intelligence on the external validation set for diagnosing lymph node metastasis in cervical cancer.


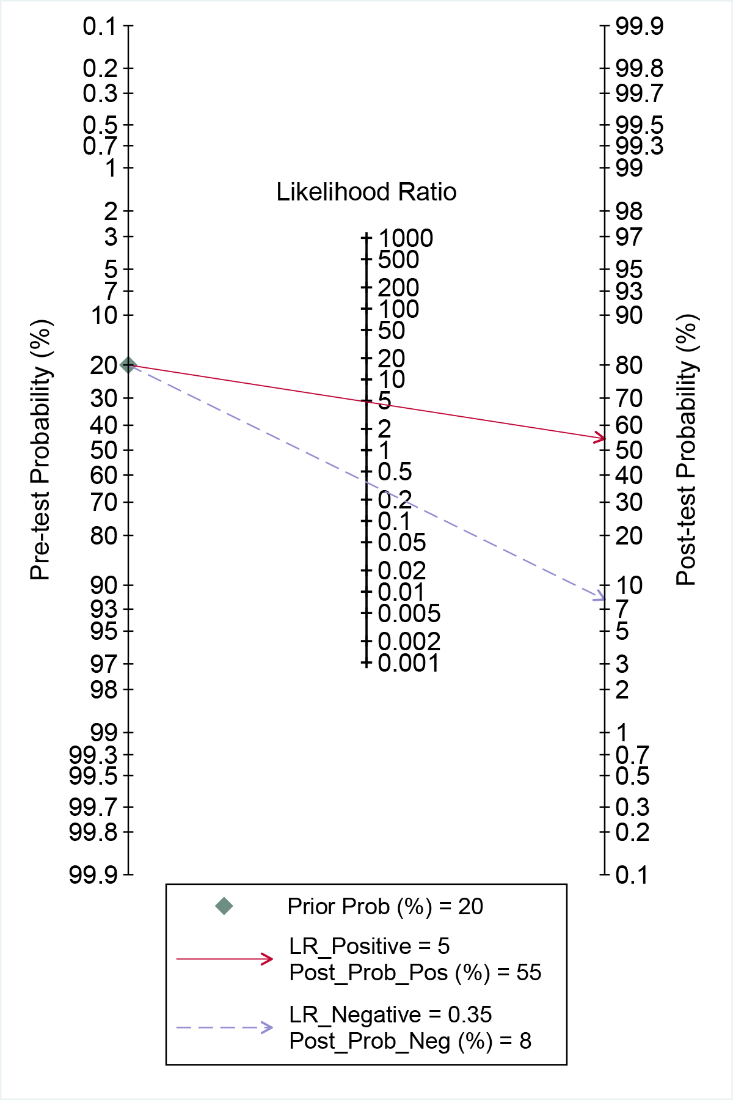


Supplementary Figure 4 Deek's funnel plot of imaging-based artificial intelligence on the external validation set for diagnosing lymph node metastasis in cervical cancer.


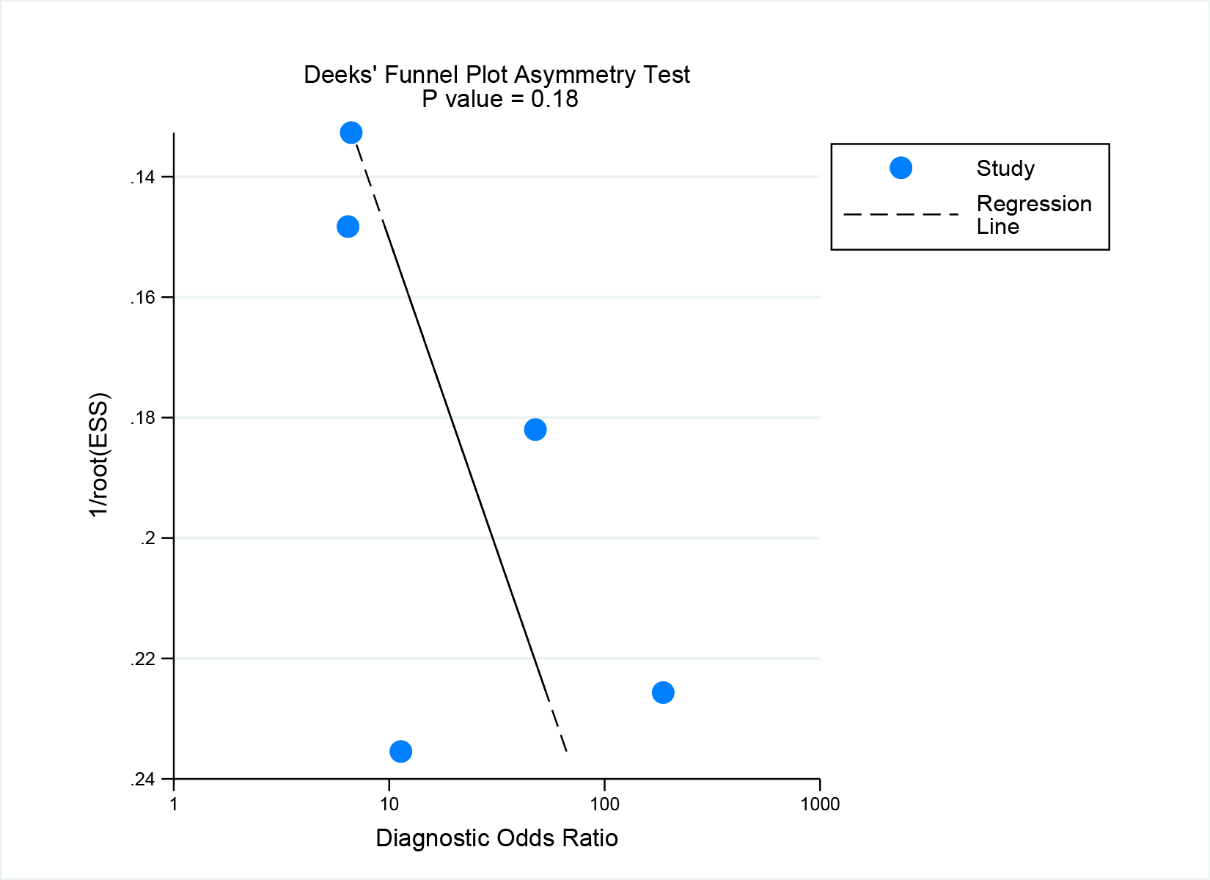

Supplement: Supplementary file 1 [file DataSheet1.docx]
